# Sea Cucumber Peptide Alleviates Ulcerative Colitis Induced by Dextran Sulfate Sodium by Alleviating Gut Microbiota Imbalance and Regulating miR-155/SOCS1 Axis in Mice

**DOI:** 10.3390/foods12183434

**Published:** 2023-09-15

**Authors:** Jing Mao, Yunjiao Zhao, Lechen Wang, Tao Wu, Yan Jin, Jing Meng, Min Zhang

**Affiliations:** 1 State Key Laboratory of Food Nutrition and Safety, College of Food Science and Engineering, Tianjin University of Science & Technology, Tianjin 300457, China; 2 School of Biological Science and Food Engineering, Chuzhou University, Chuzhou 239000, China; 3 Tianjin International Joint Academy of Biomedicine, Tianjin 300450, China; 4 China−Russia Agricultural Products Processing Joint Laboratory, Tianjin Agricultural University, Tianjin 300384, China

**Keywords:** sea cucumber peptide, ulcerative colitis, miR-155, SOCS1, gut microbiota

## Abstract

Sea cucumber peptides have been proven to exhibit a variety of biological activities. Ulcerative colitis (UC) is a chronic disease characterized by diffuse inflammation of the mucosa of the rectum and colon with increasing incidence and long duration, and is difficult to cure. The effect of sea cucumber peptide on UC is currently unknown. In this study, 1.5% dextran sulfate sodium (DSS) was added to the drinking water of mice to induce a UC model, and the daily doses of sea cucumber peptide (SP) solution of 200 mg/kg·BW, 500 mg/kg·BW, and 1000 mg/kg·BW were given to UC mice to detect the relieving effect of SP. The results showed that SP can reduce the disease activity index (DAI) of UC mice induced by DSS and can alleviate colon shortening, intestinal tissue damage, and the loss of intestinal tight junction proteins (Claudin-1, Occludin). SP decreased the spleen index, pro-inflammatory factors (IL-1β, IL-6, TNF-α), and myeloperoxidase (MPO) levels in UC mice. SP can alleviate the imbalance of gut microbiota in UC mice, increase the abundance of the *Lachnospiraceae NK4A136 group*, *Prevotellaceae UCG-001*, and *Ligilactobacillus*, and reduce the abundance of *Bacteroides* and the *Eubacterium rum group*, as well as alleviating the decrease in short-chain fatty acid (SCFA) content in the feces of UC mice. Notably, SP inhibited miR-155 expression in the colon tissue of UC mice and increased its target protein, suppressor of cytokine signaling 1 (SOCS1), which acts as an inflammatory inhibitor. In summary, the ameliorative effect of SP on UC may be achieved by improving the imbalance of gut microbiota and regulating the miR-155/SOCS1 axis. This study provides a new idea for developing SP as a nutritional supplement to maintain intestinal health.

## 1. Introduction

Inflammatory bowel diseases (IBDs), including Crohn’s disease (CD) and ulcerative colitis (UC) [1], have become a global disease in the 21st century, and their incidence in developing countries is on the rise [2,3]. IBD has the highest prevalence in Europe and North America, and UC has an incidence of 0.14–6.5 per 100,000 people in Asia [2]. UC is a chronic disease characterized by diffuse inflammation of the colonic and rectal mucosa [4]. The cause of UC is unknown, and epidemiological data suggest that genetic and environmental factors contribute to the risk of UC [5]. Patients with UC typically present with bloody diarrhea; pus, mucus, or both; and abdominal cramps during defecation [6]. The etiology of UC is unknown and cannot be cured [5]. The drugs used to treat UC include 5-aminosalicylic acid and corticosteroids, with serious side effects. When UC has complications such as intestinal perforation, toxic megacolon, and rectal bleeding, surgical treatment is required, but surgical treatment can lead to high stool frequency, fecal incontinence, and decreased fertility [6]. The common cause of death in UC patients is colorectal cancer, and the other main causes are toxic megacolon, intestinal perforation, and intestinal infarction [4]. Studies have shown that dietary interventions can help alleviate UC, so new nutritional supplements can be developed to alleviate the condition.

Sea cucumber is a kind of marine invertebrate with high nutritional value and potential health value, which is very popular in many countries in Asia [7]. The crude protein content of sea cucumber ranges from 40.7% to 63.3% [8]. Enzymatic hydrolysis and microbial fermentation are used for the production of sea cucumber peptides [9]. Bioactive peptides provide a variety of amino acids when absorbed through the intestine and are mild with no side effects [9]. Sea cucumber peptides have been reported to have immunomodulatory, hypoglycemic, hypertensive, memory-enhancing, antioxidant, and other biological activities [10,11,12,13]. Sea cucumber protein contains a variety of essential and non-essential amino acids, so sea cucumber peptides may have various biological activities [9]. In addition, hydrolytic peptides have also been shown to play a role in alleviating UC [14,15].

MicroRNAs (miRNAs) are RNA regulators of gene expression about 21 nucleotides in length, which have the effect of preventing the synthesis of target proteins [16]. miR-155 has been shown to play an important role in body immunity [17]. As a potent inflammatory inhibitor, suppressor of cytokine signaling 1 (SOCS1) can negatively regulate the JAK-STAT signaling pathway [18]. Previous research has established that miR-155 regulates the inflammatory phenotype of intestinal myofibroblasts by targeting SOCS1 in UC [19]. In this study, we investigated the mechanism of SP on UC by detecting the changes in the miR-155/SOCS1 axis.

Sea cucumber protein has been reported to relieve colitis in mice through anti-inflammatory effects and the regulation of gut microbiota [20]. In this study, DSS was used to establish a mouse model of UC to detect the ameliorative effect of SP on it. We investigated the effects of SP on UC by detecting the changes in the disease activity index (DAI), colon injury, immune organ index, immune factor level, short-chain fatty acid (SCFA) content, and gut microbiota of mice in each group. At the same time, the levels of miR-155 and SOCS1 in UC mice were detected to explore the mechanism of SP on UC from the perspective of non-coding RNA. Through the correlation analysis of gut microbiota and UC-related indicators, the key intestinal microorganisms that alleviate UC by SP and the intestinal microorganisms associated with the changes in miR-155 were found.

## 2. Materials and Methods

### 2.1. Preparation of Sea Cucumber Peptide

The sea cucumber peptide was provided by Baidefu Biotechnology Co., Ltd. (Tangshan, Hebei, China), obtained through the enzymolysis of sea cucumber (*Acaudina molpadioides*). The sea cucumbers were eviscerated and cleaned, mixed with water, and ground thoroughly using a beater. The sea cucumbers were hydrolyzed at 40 °C by protease (papain: trypsin 2:1, *w*/*w*, Novozymes, Bagsvaerd, Denmark) for 5 h. After hydrolysis, the mixture was heated at 95 °C for 20 min to inactivate the enzyme and then centrifuged to obtain the supernatant. After filtration, purification, and drying, the sea cucumber peptide was obtained.

### 2.2. Animals and Experimental Design

Male C57BL/6 mice were purchased from SiPeiFu Biotechnology Co., Ltd. (Beijing, China). The mice were raised in a sterile environment with a temperature of 20–25 °C, humidity of 45–55%, light for 12 h, and darkness for 12 h. All animal experiments in this study followed the principles established by the Animal Ethics Committee of Tianjin University of Science & Technology (approval number: TUST20210910).

Fifty healthy mice (18 ± 2 g) were divided into five groups: Normal group, DSS group, SP low-dose group (SPL), SP medium-dose group (SPM), and SP high-dose group (SPH). The experiment lasted for 7 weeks. Mice in the DSS and SP groups were given 1.5% DSS solution at 1, 3, 5, and 7 weeks, and sterile water at 2, 4, and 6 weeks. Normal mice were given sterile water throughout the experimental cycle. The intragastric doses of the SP groups were 200 mg/kg·BW, 500 mg/kg·BW, and 1000 mg/kg·BW, respectively. The Normal group and DSS group were given the same volume of sterile water every day. During the experiment, the disease activity index (DAI) was recorded every 3 days according to the previous method [14]. At the end of the experiment, the mice and spleens were weighed. The spleen index was calculated according to the following equation: Spleen index (mg/g) = spleen weight (mg)/mice weight (g). Colon contents were collected and stored in a −80 °C refrigerator. Parts of the colon were respectively stored in a −80 °C refrigerator and 4% paraformaldehyde solution to prepare for the next experiment.

### 2.3. ELISA Assay

The colonic tissue was homogenized and then the supernatant was taken by centrifuge for ELISA detection. The levels of interleukin-1β (IL-1β), interleukin-6 (IL-6), tumor necrosis factor-α (TNF-α), and myeloperoxidase (MPO) in colon homogenate were detected using an ELISA kit from the Shanghai Enzyme-linked Biotechnology Co., Ltd. (Shanghai, China).

### 2.4. Determination of SCFA Concentration

The extraction and detection of short-chain fatty acids (SCFA) in UC mice feces was conducted according to previous methods [21].

### 2.5. Histopathological Examination

Colon tissue preserved with 4% paraformaldehyde was embedded in paraffin, sectioned, and stained with hematoxylin and eosin (H&E). Pathological scores were evaluated according to the degree of tissue damage and inflammatory cell infiltration. Goblet cells were detected by alixin blue (AB) and periodic acid Schiff (PAS) staining. The pathological scores of colonic sections of the mice in each group were performed according to previous methods [22,23].

### 2.6. RT-PCR

The total RNA was extracted from mouse colon tissues with TRIzol (Biosharp, Hefei, China) reagent according to the instructions. The primers used in the experiment were synthesized by GENEWIZ Biotechnology Co., Ltd. (Suzhou, Jiangsu, China), and their sequences are shown in Table 1. Reverse transcription was performed using the first-strand cDNA Synthesis Kit (TIANGEN, Beijing, China). PCR reagents were prepared according to the instructions of the RT-PCR kit (TIANGEN, Beijing, China). Changes in the fluorescence intensity during PCR were identified and quantified using a fluorescence quantitative PCR instrument (CFX, Bio-Rad, Hercules, CA, USA).

### 2.7. Western Blot

The samples were prepared by grinding the colonic tissue of experimental mice with high-efficiency RIPA lysate (Solarbio, Beijing, China) supplemented with protease inhibitors. A total protein assay kit (Jiancheng, Nanjing, China) was used to determine the protein content of the samples. The samples were separated by polyacrylamide gel electrophoresis and transferred to a polyvinylidene difluoride membrane. The membrane was sealed with 5% skim milk powder for 1 h at room temperature. After incubation, proteins on the membrane were detected with ECL chemiluminescence substrate (Biosharp, HeFei, China) and blotted on a gel imaging system (LAS4000, GE, Boston, MA, USA). Primary antibodies against Claudin-1 (Bioss, Woburn, MA, USA, bs-1428R, 1/1000), Occludin (Bioss, bs-10011R, 1/1000), SOCS1 (Abcepta, San Diego, CA, USA, AP8790A, 1/1000), β-actin (Proteintech, Rosemont, IL, USA 20536-1-AP, 1/2000) and secondary antibody (Proteintech, SA00001-2, 1/5000) were used for Western blot analysis. The intensity of the protein bands was analyzed using image analysis software (Image J 1.53a, Bethesda, MD, USA).

### 2.8. High-Throughput Sequencing of Gut Microbiota

In this study, sequencing was used to analyze the differences in the intestinal microbiota in each group of mice. Fecal microbial genomes were extracted from the mouse colon contents, and the DNA purity and concentration were determined. The V3–V4 hypervariable region (primer: 341F: CCTAYGGGRBGCASCAG, 806R: GGACTACNNGGGTATCTAAT) of 16S rRNA was amplified using diluted genomic DNA as a template. The PCR products were detected by electrophoresis on agarose gel, and the target bands were recovered by gluing. A Thermo Scientific GeneJET Glue recovery Kit was used to purify the product. Qualified libraries were sequenced using NovaSeq 6000 (Beijing, China).

### 2.9. Statistical Analysis

All experiments were statistically analyzed using GraphPad Prism 7 (San Diego, CA, USA) and SPSS19 (Armonk, NY, USA). One-way ANOVA calculations showed statistically significant differences. The results are expressed as mean ± standard deviation. *p* < 0.05 was considered as a statistically significant difference.

## 3. Results

### 3.1. SP Alleviated DSS-Induced UC

**Figure 1:**
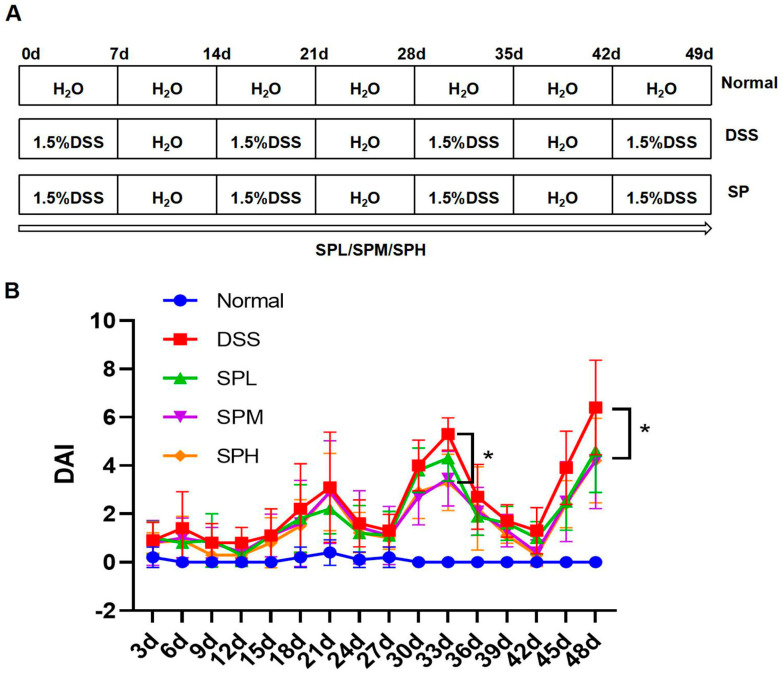
Experimental flow chart and DAI score. (**A**) Experimental design flow chart; (**B**) DAI score. Results are represented as means ± SD. * *p* < 0.05 in a comparison with DSS group.

DSS-induced UC in mice is characterized by weight loss, bloody diarrhea, ulceration, epithelial cell loss, and neutrophil infiltration, similar to the symptoms of human UC. Based on this, the DSS-induced UC model was used to explore the ameliorative effect of SP on UC. The experimental flow chart is shown in Figure 1A. The DAI score was used to evaluate the protective effect of SP on DSS-induced UC. In the DSS group, DAI increased significantly on day 21 and symptoms worsened significantly on day 33. On day 33, the DAI score in the SPM and SPH groups were significantly lower than that in the DSS group (*p* < 0.05). On day 48, the DAI scores of the SP groups were significantly lower than that of the DSS group (*p* < 0.05), indicating that SP can alleviate the symptoms of UC (Figure 1B).

### 3.2. SP Alleviated DSS-Induced Colon Tissue Injury

**Figure 2:**
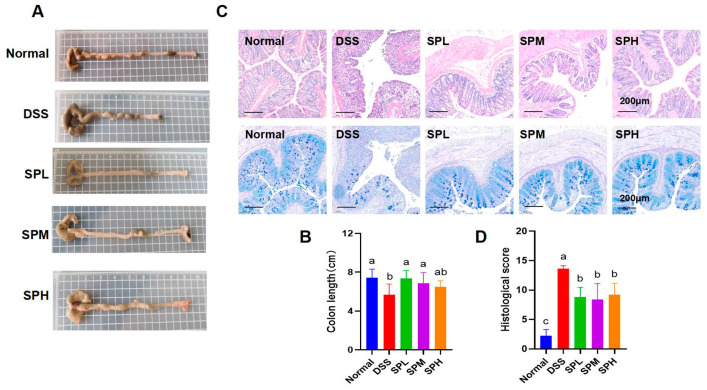
SP alleviated intestinal injury in mice with UC. (**A**) Images of colon tissue; (**B**) colon length; (**C**) colon H&E staining and AB-PAS staining of UC mice; (**D**) colon histopathological score. Results are represented as means ± SD. Different lowercase letters indicate a significant difference.

The colon length of the mice was measured. Representative images of the colons in each group are shown in Figure 2A. The shortening of the colon is an important index to evaluate the severity of UC. In Figure 2B, we can see that the colon length in the SPL and SPM groups is significantly higher than that in the DSS group (*p* < 0.05). Next, we observed the degree of colonic tissue injury and the number of goblet cells by staining the colonic tissue sections of the mice. Goblet cells are involved in mucosal defense and the repair of intestinal epithelial cells, thereby helping to maintain intestinal barrier integrity. In the normal group, the colon tissue was in good condition, the intestinal epithelium and crypts were arranged in a complete and orderly manner, a large number of goblet cells were seen, and there was no inflammatory infiltration. However, in the DSS group, a large number of intestinal epithelial cells were necrotic, mucosal, and submucosal in terms of inflammatory infiltration, the goblet cells were significantly reduced, crypt injury was severe, and the pathological score was higher. Compared with the DSS group, the number of goblet cells in the SP groups were higher (Figure 2C). Compared with the DSS group, colonic injury was significantly reduced in the SP groups, and the pathological scores were significantly lower than that in the DSS group (*p* < 0.05) (Figure 2C,D). To sum up, SP can effectively alleviate colon injury caused by DSS.

**Figure 3:**
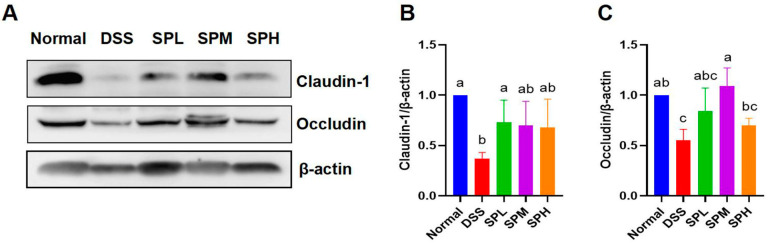
Effect of SP on tight junction proteins in UC mice. (**A**) Protein bands of Claudin-1 and Occludin; (**B**) Relative expression of Claudin-1; (**C**) relative expression of Occludin. Results are represented as means ± SD. Different lowercase letters indicate a significant difference.

Tight junction proteins help maintain intestinal barrier integrity, thereby contributing to gut health. The contents of Claudin-1 and Occludin were determined (Figure 3A–C). Compared with DSS group, Claudin-1 content in the SPL group was significantly higher (*p* < 0.05), and the Occludin content in the SPM group was significantly higher (*p* < 0.05).

### 3.3. Anti-Inflammatory Effects of SP

**Figure 4:**
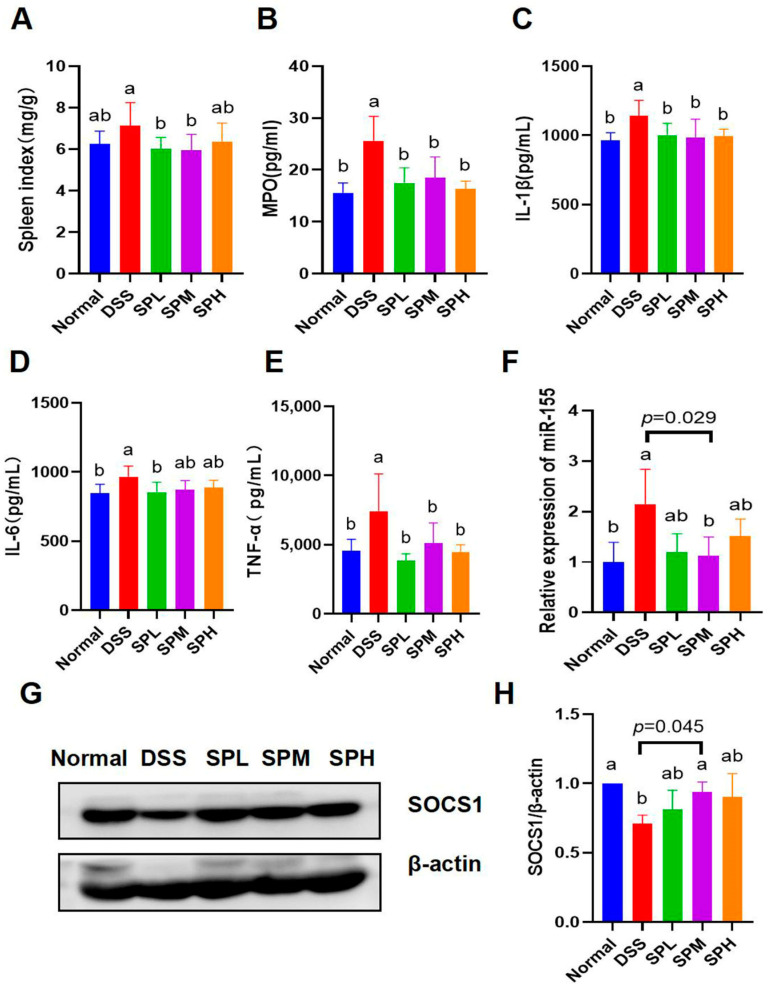
Anti-inflammatory effect of SP on UC mice. (**A**) Spleen index; (**B**) MPO; (**C**) IL-1β; (**D**) IL-6; (**E**) TNF-α; (**F**) relative expression of miR-155; (**G**) protein bands of SOCS1; (**H**) relative expression of SOCS1. Results are represented as means ± SD. Different lowercase letters indicate a significant difference.

The spleen is an important immune organ, and when the body is stimulated, immune cells such as dendritic cells, T cells, and B cells proliferate in the spleen, resulting in splenomegaly [24]. Therefore, the spleen index was used to evaluate the inflammatory status of UC mice. Figure 4A showed that the spleen index in the SPL and SPM groups was significantly lower than that in the DSS group (*p* < 0.05). In addition, myeloperoxidase (MPO) and three representative proinflammatory factors were measured. The contents of colonic homogenate MPO, IL-1β, IL-6, and TNF-α in the DSS group were significantly higher than those in the normal group (*p* < 0.05). Compared with the DSS group, the MPO level in the SP groups, IL-1β level in the SP groups, IL-6 level in the SPL group, and TNF-α level in the SP groups were significantly decreased (*p* < 0.05) (Figure 4B–E). These results indicate that SP has anti-inflammatory effect.

RT-qPCR was used to detect the expression level of miR-155 in the colon tissues of mice. The results showed that the content of miR-155 in the SPM group was significantly lower than that in the DSS group (*p* < 0.05) (Figure 4F). In addition, the expression levels of SOCS1 protein in colon tissues were detected using Western blot. Compared with the DSS group, the SOCS1 protein content was significantly higher in the SPM group (*p* < 0.05) (Figure 4G,H).

### 3.4. Effect of SP on the Content of SCFA in Mouse Feces

**Figure 5:**
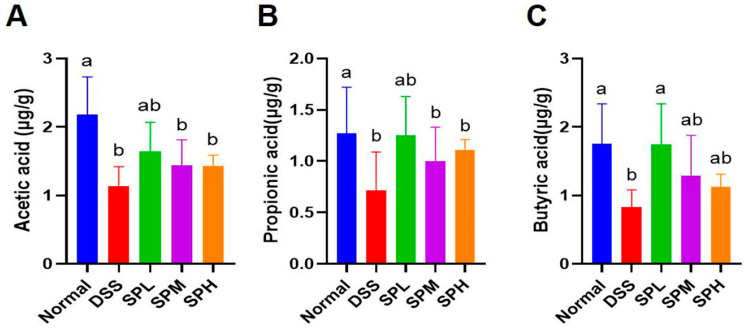
Effect of SP on the content of SCFA in feces of UC mice. (**A**) Acetic acid; (**B**) propionic acid; (**C**) butyric acid. Results are represented as means ± SD. Different lowercase letters indicate a significant difference.

Butyric acid and other SCFAs are the fermentation products of intestinal microorganisms, which have an influence on body health. The content of SCFAs in mouse feces was determined by gas chromatography (Figure 5A–C). Compared with the DSS group, the contents of butyric acid in the SPL group were significantly higher (*p* < 0.05).

### 3.5. SP Regulates the Gut Microbiota

**Figure 6:**
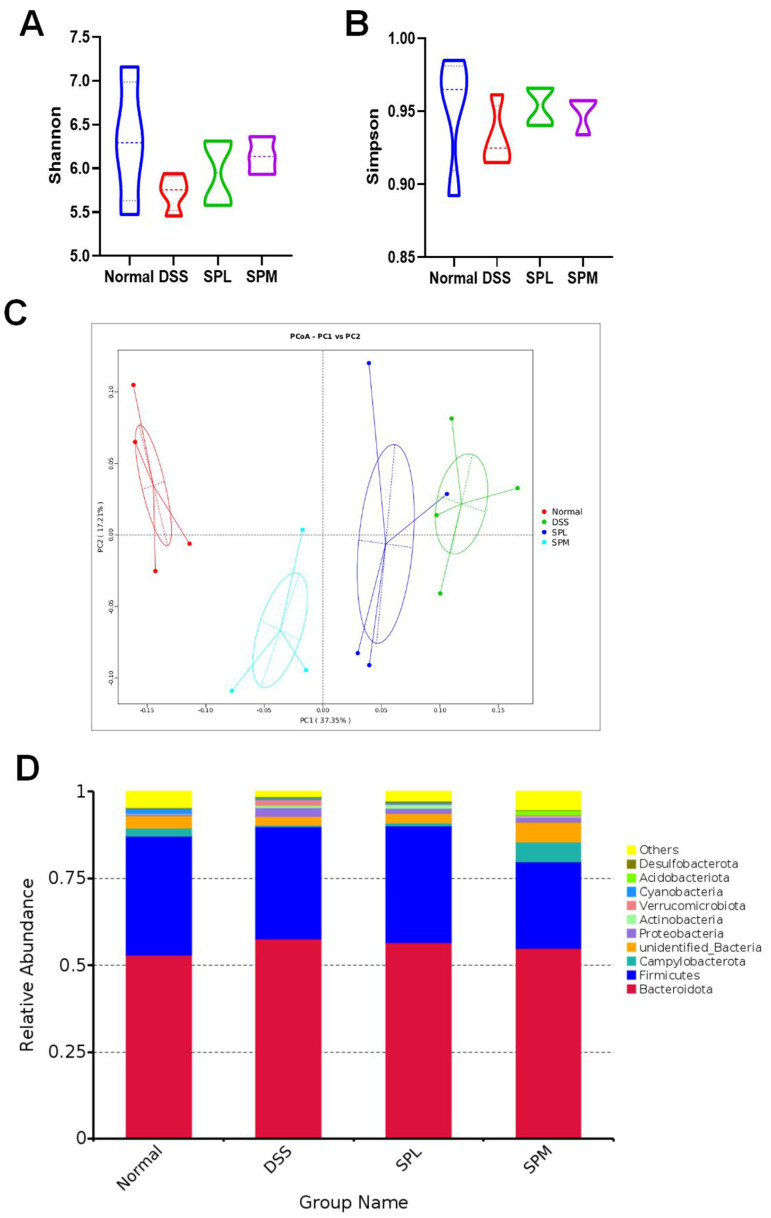
Regulation of SP on gut microbiota. (**A**) Shannon index; (**B**) Simpson index; (**C**) PCoA; (**D**) differences in microbiota at phylum level. Results are represented as means ± SD.

**Figure 7:**
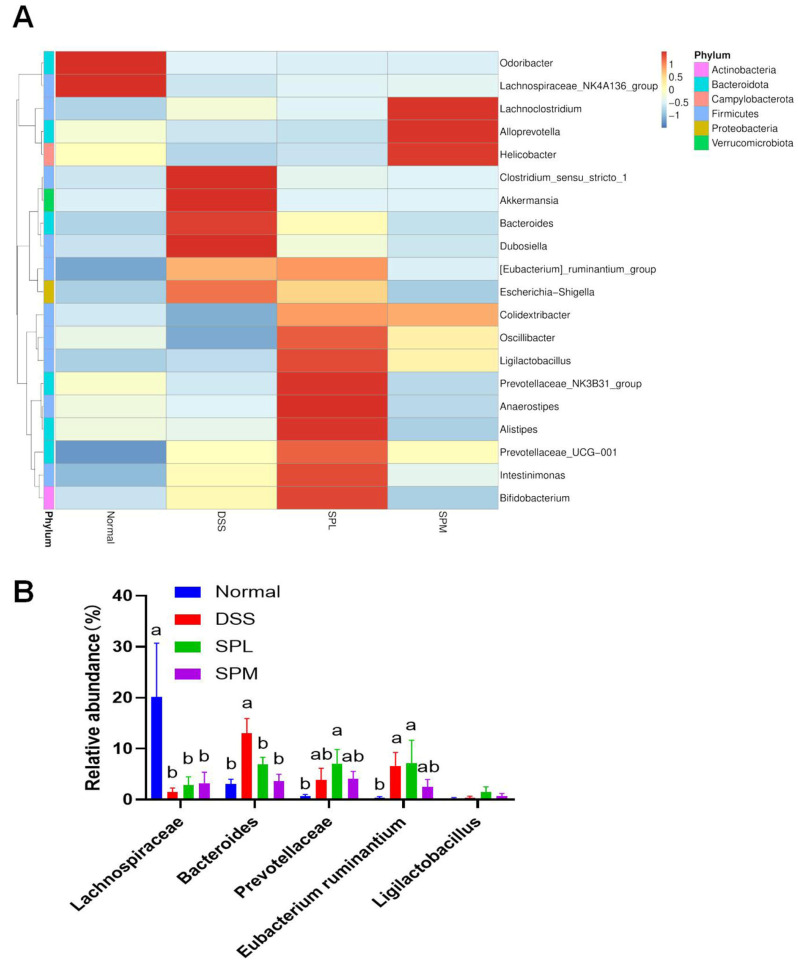
Difference in microbe abundance at genus level among all groups. Clustering heat map (**A**), differences in relative abundance of microbiota (**B**). Different lowercase letters indicate a significant difference.

**Figure 8:**
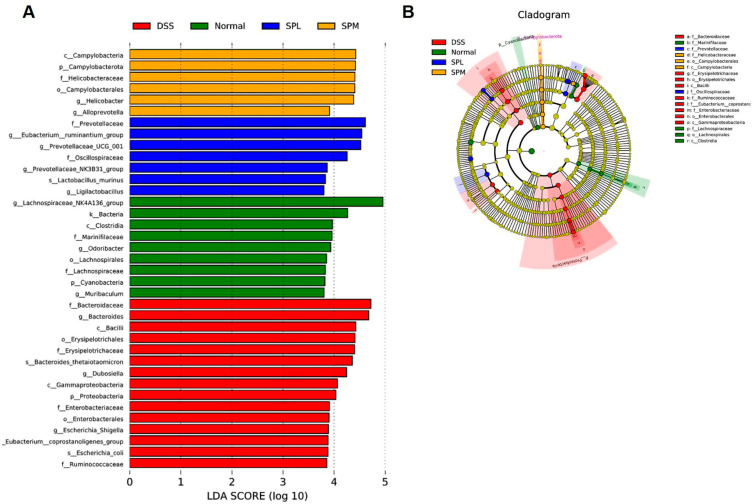
LEfse analysis of SP regulation of gut microbiota. (**A**) Distribution histogram based on LDA; (**B**) LEfSe branching diagram of evolution.

Standardized 16SrDNA sequencing was performed on the colon contents to analyze the differences in the gut microbiota in each group. Considering that the SPL and SPM groups had better alleviating effects on UC, we selected the Normal group, DSS group, and the above two groups for gut microbiota sequencing analysis. To evaluate the effect of SP on intestinal microbial diversity, α diversity analysis was used. In Figure 6A,B, the Shannon and Simpson indices show that the intestinal microbial diversity of mice in the DSS group was reduced compared with normal mice, and SP intervention can reverse this trend. In addition, principal coordinate analysis (PCoA) showed that the DSS group was significantly different from the normal group, and SP alleviated the intestinal microbiota imbalance caused by DSS (Figure 6C). As shown in Figure 6D, Bacteroidetes and Firmicutes were the main phyla in the gut microbiota of the mice in each group. Compared with the normal group, the Bacteroidetes increased and Firmicutes decreased in the DSS group, and this trend was alleviated in the SPL group. Proteobacteria increased significantly in DSS group, and SP intervention reversed the increase. The cluster heat map can directly show the relatively abundant microflora in the gut of each group of mice (Figure 7A). *Odoribacter* and *Lachnospiraceae NK4A136 group* had high relative abundance in the intestines of normal group mice. The relative abundance of *Clostridium sensu stricto 1*, *Akkermansia*, *Bacteroides*, and *Dubosiella* was higher in the intestines of the DSS group mice. The relative abundance of *Ligilactobacillus*, *Prevotellaceae NK3B31 group*, *Anaerostipes*, *Alistipes*, *Prevotellaceae UCG-001*, *Intestinimonas*, and *Bifidobacterium* was higher in the intestinal tract of SPL group mice. The dominant microflora in the SPM group were *Lachnoclostridium*, *Alloprevotella*, and *Helicobacter*. The differences in the intestinal microflora at genus level among the groups are shown in Figure 7B. Compared with the DSS group, the relative abundances of *Bacteroides* and the *Eubacterium ruminantium group* decreased, while the abundances of the *Lachnospiraceae NK4A136 group*, *Prevotellaceae UCG*-*001*, and *Ligilactobacillus* increased. LEfSe (LDA Effect Size) analysis also confirmed the changes in gut microbiota among the groups (Figure 8A,B). The predominant gut microbiota of the normal mice was the *Lachnospiraceae NK4A136 group*. In the DSS group, the relative abundance of *Bacteroidaceae*, *Erysipelotrichaceae*, and *Proteobacteria* were relatively high. The abundance of *Prevotellaceae* in the SPL group and *Alloprevotella* in the SPM group were higher.

### 3.6. Correlation between the Gut Microbiota and UC

**Figure 9:**
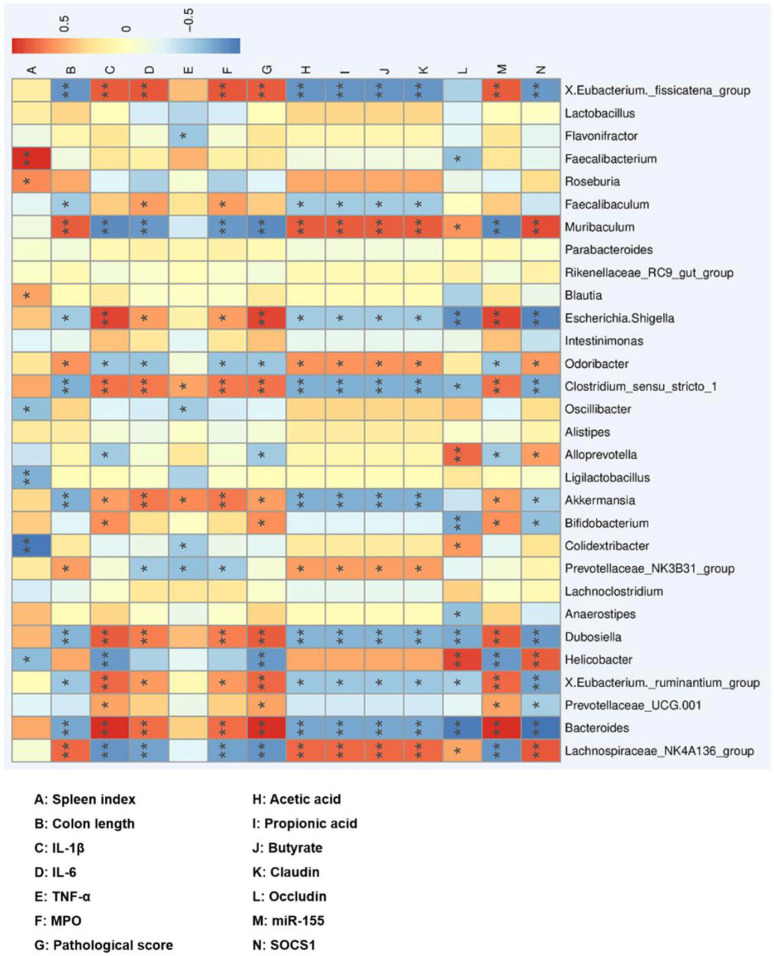
Correlation analysis of intestinal microbiota and UC-related indexes. Blue: negative correlation, red: positive correlation; * *p* < 0.05, ** *p* < 0.01.

Spearman correlation analysis was performed between the relative abundance of gut microbiota affected by SP and UC parameters to identify the key intestinal microbiota that may alleviate UC after SP intervention (Figure 9). *Bacteroides*, *Eubacterium ruminantium group*, *Dubosiella*, *Bifidobacterium*, *Akkermansia*, *Clostridium sensu stricto 1*, *Escherichia. Shigella*, *Eubacterium fissicatena group,* and *Turicibacter* were positively correlated with UC severity, while *Lachnospiraceae NK4A136 group* and *Muribaculum* were negatively correlated.

## 4. Discussion

The incidence rate of UC in the world is on the rise, which seriously affects the health and quality of life of patients. Sea cucumber peptides have been reported to exhibit a variety of biological activities. Egg white peptides have been reported to alleviate DSS-induced UC in mice by inhibiting pro-inflammatory cytokines and regulating intestinal microbiota imbalances [15]. Matsutake-derived peptides may also alleviate DSS-induced UC by alleviating intestinal damage and inhibiting the NF-κB pathway [14]. Bioactive peptides regulate intestinal homeostasis, including the mucosal immune response, inflammatory response, and intestinal microbiota [25]. In this study, a DSS-induced UC model was used to investigate the effect of SP on UC in mice. The results showed that after the oral administration of SP in UC mice, the symptoms of bloody stool, diarrhea, colon shortening, intestinal wall integrity reduction, and inflammatory cell infiltration were relieved. Therefore, SP is expected to be a nutritional supplement that can alleviate UC.

The intervention of SP significantly reduced the intestinal tissue damage and increased the number of goblet cells in UC mice. Goblet cells contribute to the mucosal defense and repair of intestinal epithelial cells, and mucus proteins synthesized by goblet cells are the main components of the intestinal mucus layer, whose function is to protect epithelial cells [26]. Intestinal barrier dysfunction is a major feature of UC [27]. Intestinal epithelial barrier dysfunction can widely activate the mucosal immune response and accelerate the occurrence and severity of UC [28]. Epithelial cells play a key role as a barrier against external factors, and tight junction proteins are essential for maintaining the integrity of the epithelial barrier [29,30]. SP can effectively alleviate tight junction protein loss and help maintain the intestinal epithelial barrier. In short, SP ameliorates UC by alleviating intestinal injury.

The spleen index, the contents of MPO, and three proinflammatory factors of UC mice were decreased by SP intervention, suggesting that SP has an anti-inflammatory effect. The spleen index is usually used to evaluate the body’s immune level, and when the spleen index is too high, it indicates an inflammatory reaction. Cytokines are directly involved in the pathogenesis of UC and are closely related to the severity of UC. IL-1β, IL-6, and TNF-α are all proinflammatory factors, which are closely related to the pathogenesis of UC. The infiltration of neutrophils and macrophages in colon tissue is related to the severity of inflammation. Myeloperoxidase (MPO) can be used as an indicator of neutrophil infiltration to evaluate the severity of disease [31]. These results indicate that SP has anti-inflammatory effects and can alleviate UC in mice.

The miR-155/SOCS1 axis is involved in the anti-inflammatory effect of SP on UC mice. miR-155 is a miRNA in the immune system, and the high expression of miR-155 contributes to the occurrence of chronic inflammation, autoimmunity diseases, and cancer [32]. miR-155 affects the severity of UC by regulating immune-related proteins or immune cells [33,34]. Studies have shown that the regulation of miR-155 in colitis may be related to the regulation of TH17 [35]. miR-155 has been reported to promote colitis-associated intestinal fibrosis, targeting the HBP1/Wnt/β-catenin signaling pathway [33]. Excessive or dysregulation of cytokine signaling can lead to a variety of diseases. SOCS1 plays an important role in regulating the immune system. Previous studies have reported that SOCS1 negatively regulates the JAK-STAT pathway by inhibiting the activity of JAK tyrosine kinase [18]. SOCS1 has a negative regulatory effect on T cells and cytokines [36]. Previous studies have confirmed that miR-155 is abnormally expressed in UC patients and regulates inflammation by targeting SOCS1 protein [19]. Consistent with previous studies, the decrease in the inflammatory state in the SP groups of mice was accompanied by the decrease in miR-155 and the increase in SOCS1. The regulation of SP on the miR-155/SCOS1 axis suggests that SP exerts anti-inflammatory effects on UC in mice through non-coding gene targets.

SCFAs mainly include acetic acid, propionic acid, and butyric acid, which are the products of intestinal microbial fermentation. Studies have shown that lactic acid bacteria can regulate the gut microbial community and reduce the abundance of harmful bacteria by synthesizing SCFAs [37]. Previous studies have shown that the supplementation of SCFA helps to improve diseases such as hyperglycemia and obesity [38,39,40]. Similarly, in our experiment, SP also showed the effect of increasing the content of SCFA. The change in the SCFA content was related to the abundance of some microorganisms in the gut microbiome, indicating that SP changed the gut microbiome of UC mice.

Gut microbiota imbalance is considered to be one of the causes of UC. Extensive data show that the gut microbiota of DSS-induced UC mice is dysregulated. The increase in *Proteobacteria* is a sign of intestinal microbiota imbalance [41]. In this study, *Proteobacteria* increased in the DSS group and decreased after SP intervention. Studies have shown that *Bacteroides* is associated with the development of UC and inversely correlated with SCFA content [42]. *Lachnospiraceae* hydrolyze starch and other sugars to produce butyric acid and other SCFAs [43]. Therefore, the increase in the SCFA content in the SP groups may be related to the changes in the abundance of the above intestinal microorganisms. Previous studies have shown that the abundance of *Prevotellaceae* in the intestinal tract of UC mice is decreased [44], and it is negatively correlated with pro-inflammatory cytokines [15]. Therefore, SP has anti-inflammatory effects by increasing the abundance of *Prevotellaceae*. Consistent with previous results, SP alleviates UC by regulating the abundance of *Erysipelotrichaceae* [45], *Escherichia Shigella* [42], *Eubacterium ruminantium group* [46], *Clostridium sensu stricto 1* [47], and *Turicibacter* [48]. SP intervention changed the gut microbiota structure and the relative abundance of specific microbiota in the UC mice, decreased the abundance of harmful microorganisms, and increased the abundance of SCFA-producing microbiota. Therefore, SP can improve UC in mice by regulating the gut microbiota.

In this study, we demonstrated that SP intervention significantly improved DSS-induced UC. The addition of SP resulted in significant changes in the overall structure of gut microbiota and specific intestinal bacteria, which were significantly correlated with the expression of UC-related symptoms, inflammatory factors, SCFAs, and tight junction proteins.

## 5. Conclusions

Our study shows that SP can effectively improve DSS-induced UC in mice. SP can reduce the DAI index and intestinal injury in UC mice, reduce inflammation levels by reducing the spleen index, pro-inflammatory factor level, and the regulation of the miR-155/SOCS1 axis, alleviate gut microbiota imbalance by changing the gut microbiota structure and abundance of specific flora in UC mice, and alleviate SCFA reduction. The changes in the microflora abundance in the gut of UC mice were closely correlated with the DAI index, colon pathological score, tight junction protein content, pro-inflammatory cytokine content, and the miR-155/SOCS1 axis and other UC indicators. In summary, SP mainly alleviates UC in mice by reducing inflammation levels and alleviating gut microbiota imbalance. The beneficial effect of SP on DSS-induced UC suggests that SP is a functional food worthy of development.

## Figures and Tables

**Table 1:** Primers used for RT-qPCR.

Gene Symbol	Primer	Primer Sequence (5′-3′)
miR-155	RT primer	GTCGTATCCAGTGCGTGTCGTGGAGTCGGCAATTACCCCTAT
	Forward	TGCGGTTAATGCTAATTGTGA
	Reverse	CAGTGCGTGTCGTGGAGT
U6	RT primer	AAAATATGGAACGCTTCACGA
	Forward Reverse	CGCTTCGGCAGCACATATAC AAAATATGGAACGCTTCACGA

## Data Availability

The data presented in this study are available on request from the corresponding author.
